# Relationship between Serum Alkaline Phosphatase and Low Muscle Mass Index Among Korean Adults: A Nationwide Population-Based Study

**DOI:** 10.3390/biom11060842

**Published:** 2021-06-05

**Authors:** Jun-Hyuk Lee, A-Ra Cho, Yong-Jae Lee

**Affiliations:** 1 Department of Family Medicine, Nowon Eulji Medical Center, Eulji University School of Medicine, Seoul 01830, Korea; swpapa@eulji.ac.kr; 2 Department of Family Medicine, Yong-in Severance Hospital, Yonsei University College of Medicine, Yong-in 16995, Korea; ARA1713@yuhs.ac; 3 Department of Family Medicine, Gangnam Severance Hospital, Yonsei University College of Medicine, Seoul 06273, Korea

**Keywords:** alkaline phosphatase, sarcopenia, inflammation

## Abstract

Sarcopenia has attracted interest due to its impact on various health problems. Chronic inflammation is an important contributor to sarcopenia. Thus, we aimed to investigate the association between serum alkaline phosphatase (ALP), which is a novel inflammatory marker, and muscle mass. This study included 15,579 adults from the 2008–2011 Korea National Health and Nutrition Survey. Low skeletal muscle mass index (LSMI) was defined as body mass index-adjusted appendicular skeletal muscle mass less than 0.789 for men and 0.512 for women. Multiple logistic regression revealed that the highest ALP tertile was significantly associated with LSMI compared with the lowest ALP tertile in both men [Odds ratio (OR): 1.41; 95% confidence interval (CI): 1.04–1.91] and women (OR: 1.45; 95% CI: 1.00–2.10) after adjusting for other confounders. On the receiver operating characteristic curve analysis, the predictive power was significantly higher for ALP levels than for white blood cell count in women (*p* < 0.001), whereas the difference was not significant in men (*p* = 0.515). Our findings suggest the potential use of serum ALP as an inflammatory marker and a predictor of sarcopenia.

## 1. Introduction

Sarcopenia is a progressive and generalized skeletal muscle disorder, characterized by low muscle strength, low muscle quantity, and low physical performance [1]. Sarcopenia was first described as a decrease in muscle mass associated with normal aging in the 1980s [2]. However, it has since been recognized as an independent disease, and was included in the International Classification of Disease-10 code in 2016 [3]. Sarcopenia is well known to be associated with frailty, functional decline, and falls in elderly [4]. Recently, emerging evidence has been presented that sarcopenia is also associated with a high risk for developing cardiometabolic disorders, such as insulin resistance, type 2 diabetes, and cardiovascular disease [5,6,7]. These associations could be explained by the multifaceted pathophysiologic causes of sarcopenia, including physical inactivity, inflammation, oxidative stress, hormonal changes, and mitochondrial dysfunction [8]. Since sarcopenia is no longer a non-modifiable disease, there has been an increased interest in preventing and predicting sarcopenia rather than focusing on diagnosis and treatment.

Alkaline phosphatase (ALP) is an enzyme present in various tissues throughout the body, and most ALP in the serum is derived from the liver, bone, and intestine [9]. Thus, elevated serum ALP has been traditionally used as a marker of hepatobiliary disease or bone-related disorders. Several recent studies have suggested that serum ALP could be a novel biomarker for predicting cardiovascular disease [10,11,12]. In 2014, a meta-analysis reported that serum ALP levels are positively and independently associated with cardiovascular risk [13]. Although the direct mechanism has not been fully elucidated, vascular calcification [14] and inflammation [15,16] may be the common link between ALP and cardiovascular disease development.

Based on the available literature, we hypothesized that higher serum ALP is associated with sarcopenia. However, the usefulness of ALP as an inflammatory marker for predicting sarcopenia is not yet well understood. Therefore, we aimed to investigate the association between serum ALP levels and skeletal muscle mass using a representative sample of Korean adults.

## 2. Materials and Methods

### 2.1. Study Population

The Korean National Health and Nutrition Examination Survey (KNHANES) is a nationwide, representative, and population-based survey that is conducted annually by the Korea Centers for Disease Control and Prevention (KCDC) to monitor the health and nutritional status of the Korean population [17]. Sampling is designed according to cross-sectional, multi-stage, stratified probability based on sex, age, and geographic area. Sampling weights were assigned to each participant to generalize the units for representing the Korean population [18].

**Figure 1:**
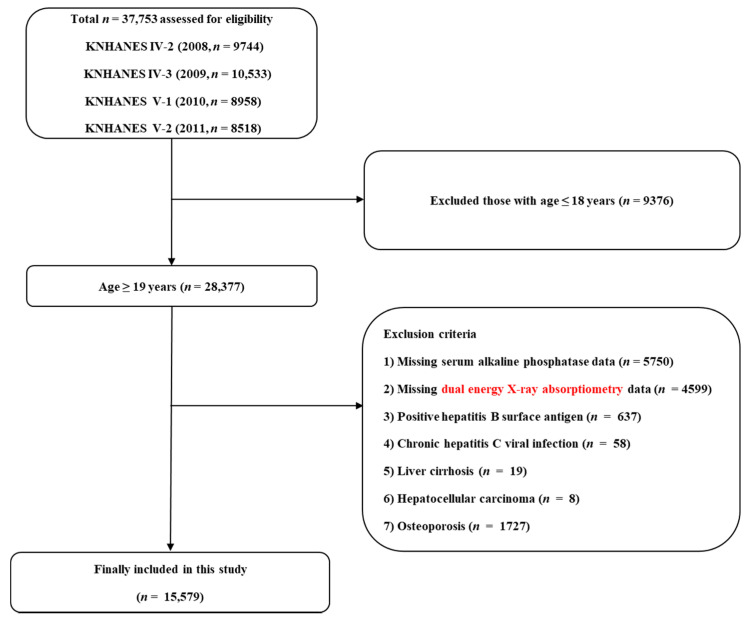
Flowchart of the study population selection.

Figure 1 shows the process of the study population selection. From a total of 37,753 people who participated in the 2008–2011 KNHANES, we included 28,377 adults over 19 years of age. Then, we excluded subjects meeting any of the following criteria: missing serum ALP data (*n* = 5750); missing dual-energy X-ray absorptiometry (DEXA) data (*n* = 4599); chronic hepatitis B viral infection (*n* = 637); chronic hepatitis C viral infection (*n* = 58); liver cirrhosis (*n* = 19); hepatocellular carcinoma (*n* = 8); patients with osteoporosis (*n* = 1727). After exclusion, a total of 15,579 participants (7171 men and 8408 women) were included in the final analysis.

### 2.2. Biochemical Measurements

Each participant’s blood sample was collected from the antecubital vein after at least 8 h of fasting. Serum ALP, total cholesterol, and fasting plasma glucose (FPG) levels were measured using a Hitachi 7600 Analyzer (Hitachi Co., Tokyo, Japan). Whole-blood white blood cell (WBC) counts were analyzed using a XE_2100D blood cell counter. The participants were classified into three groups according to the sex-specific tertiles of serum ALP levels as follows: T1, ≤ 200; T2, 201–248; T3, ≥ 249 (IU/L) for men, and T1, ≤ 170; T2, 171–224; T3, ≥ 225 (IU/L) for women.

### 2.3. Assessment of Low Skeletal Muscle Mass Index

During the 2008–2011 KNHANES, body composition data were collected from the head, arms, legs, trunk, pelvic region, and whole body by using whole-body DEXA (QDR 4500A; Hologic Inc., Bedford, MA, USA). For each anatomical region, bone mineral content (g), bone mineral density (g/cm^2^), fat mass (g), lean body mass (g), and total fat percentage (fat mass/total mass × 100) were recorded. We calculated skeletal muscle mass as lean body mass (g) − bone mineral content (g). Appendicular skeletal muscle mass (ASM) was calculated by summation of skeletal muscle mass from both arms and legs. Low skeletal muscle mass index (LSMI) was defined as a body mass index (BMI)-adjusted ASM of less than 0.789 for men and 0.512 for women, according to the Foundation for the National Institutes of Health (FNIH) Sarcopenia Project criteria [19].

### 2.4. Covariates

Height (m) and weight (kg) were measured to the nearest 0.001 m and 0.1 kg. Waist circumference (cm) was measured in the horizontal plane midway between the iliac crest and lowest rib. BMI was calculated by dividing weight by square of height (k/m^2^). A BMI less than 18.5 kg/m^2^ was considered underweight, and a BMI over 25 kg/m^2^ was considered obese, according to the definition of the Korean Society for the Study of Obesity [20]. After at least 30 min of resting, blood pressure was measured in a sitting position. Mean blood pressure (MBP; mmHg) was calculated using the following formula:

MBP = (systolic blood pressure + 2 × diastolic blood pressure)/3.(1)

Smoking status was divided into three categories: current smoker, past smoker, and never smoker. Current smokers were defined as adults who have smoked at least 100 cigarettes in their lifetime and currently smoke cigarettes. We defined past smokers as adults who had smoked at least 100 cigarettes in their lifetime but quitted smoking at the time of the survey. Never smokers were defined as adults who never smoked or smoked less than 100 cigarettes in their lifetime [21]. Heavy alcohol drinkers were defined as consuming more than 4 drinks on any day or more than 14 drinks per week for men, and consuming more than 3 drinks on any day or more than 7 drinks per week for women, based on the definition by National Institute on Alcohol Abuse and Alcoholism [22]. Physical activity was assessed by the International Physical Activity Questionnaire. Regular exercise was defined as ≥20 min of vigorous exercise for ≥3 days a week, or ≥30 min of moderate exercise/walking for ≥5 days a week. Chronic diseases included the following six comorbid conditions: diabetes mellitus, myocardial infarction, stroke, chronic obstructive lung disease, chronic kidney disease stage from 3 to 5, and any history of cancer, considering the components of the Charlson comorbidity index [23]. Participants were categorized into three groups based on these comorbidities: zero, one, or at least two chronic diseases. Total calorie intake (kcal/day) was calculated using a 24-h dietary recall method.

### 2.5. Statistical Analysis

We applied sampling weights to the participants to examine the Korean population representative data. The weights were adjusted with the values for the inverse of the response rates and the inverse of the selection probability to the sex- and age-specific values for the Korean population (post-stratification) [17].

All data analyzed in this study are presented as a mean ± standard error (SE) or percentage (SE), rather than a mean ± standard deviation, using complex survey design analysis by incorporating sample weights, stratification, and clustering. Weighted generalized linear regression analysis was used to compare differences of continuous variables among groups. For categorical variables, weighted chi-square tests were used. The odds ratios (OR) and 95% confidence intervals (CI) for LSMI of sex-specific T2 and T3 compared to T1 as the reference were calculated by using weighted multiple logistic regression analysis after adjusting for potential confounding variables. Subgroup analyses by age, BMI, alcohol intake, and physical activity were performed. To compare the ability of serum ALP levels and whole-blood WBC counts to detect the presence of LSMI, we calculated receiver operating characteristic (ROC) curves and compared the areas under the curves (AUC). All statistical analyses were conducted using SPSS statistical software (version 23.0; SPSS Inc., Chicago, IL, USA). The significance level was set at *p* less than 0.05.

## 3. Results

### 3.1. Clinical Characteristics of the Study Population

Table 1 presents the clinical characteristics of the study population. Waist circumference, MBP, FPG and the proportion of people with one or more chronic diseases were significantly increased in both men and women in the sex-specific T3 level compared to T1. Only in women, BMI and total cholesterol were significantly increased in T3 compared to T1, whereas heavy alcohol drinkers were significantly decreased. Only in men, current smokers were significantly increased, and heavy alcohol drinkers were decreased in T3 vs. T1. ASM was significantly lower in men in T3 vs. T1. Both in men and women, BMI-adjusted ASM was significantly lower in T3 vs. T1.

**Figure 2:**
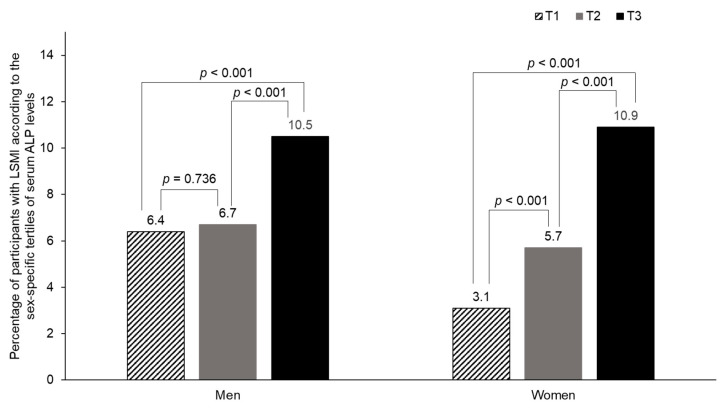
Percentage of participants with LSMI according to the sex-specific tertiles of serum ALP levels.

Figure 2 shows the percentage of participants with LSMI according to the sex-specific tertiles of serum ALP levels. In men, the prevalence of LSMI was 6.4%, 6.7%, and 10.5% in T1, T2, and T3, respectively. In women, the prevalence of LSMI was 3.1%, 5.7%, and 10.9% in T1, T2, and T3, respectively. Compared with T1, the prevalence of LSMI was significantly higher in T3 in men, and in T2 and T3 in women.

### 3.2. Relationship Between Serum ALP Level and LSMI

Table 2 shows the results of weighted multivariable logistic regression analysis of the relationship between serum ALP level and LSMI. The unadjusted ORs (95% CIs) for LSMI of T2 and T3 compared with the T1 reference were 1.05 (0.81–1.36) and 1.72 (1.35–2.20) in men and 1.88 (1.35–2.60) and 3.84 (2.81–5.24) in women, respectively. After adjusting for age, BMI, heavy alcohol drinker, smoking status, physical activity, total calorie intake, and number of chronic diseases, similar trends were observed. The fully adjusted ORs for LSMI of T3 were significantly higher compared with T1 in both men (OR: 1.41; 95% CI: 1.04–1.91) and women (OR: 1.45; 95% CI: 1.00–2.10).

**Figure 3:**
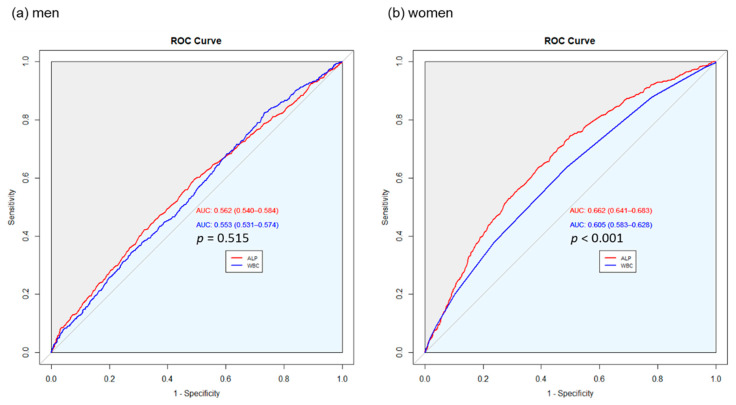
Comparison of the predictability of serum ALP levels and whole blood WBC counts for the presence of LSMI in (**a**) men and (**b**) women.

Figure 3a,b compares the predictability of serum ALP levels and whole blood WBC counts for the presence of LSMI in men and women. Despite the relatively low AUC, we found that the predictive power was significantly higher for ALP level than for WBC count in women (AUC of ALP: 0.662, AUC of WBC: 0.605, *p* < 0.001), whereas the difference was not statistically significant in men (AUC of ALP: 0.562, AUC of WBC: 0.553, *p* = 0.515).

## 4. Discussion

We examined the association between serum ALP levels and low muscle mass in a representative sample of Korean adults. Our results showed that serum ALP levels were positively associated with LSMI after adjusting for potential confounding variables in both men and women. However, the subgroup analyses showed significant associations only in the regular exercise subgroups of both men and women, the subgroups of aged over 65 and non-heavy alcohol drinkers in men, and the subgroup of women under the age of 65, probably due to the effects of other confounding variables. When comparing the predictive power for LSMI with WBC count, a representative inflammatory marker, the predictive power of serum ALP level was significantly higher in women. The predictive power for LSMI of serum ALP level was not inferior to that of WBC count in men.

Our findings are consistent with previous studies suggesting a link between sarcopenia and inflammation or inflammatory markers. Several studies have emphasized the importance of inflammatory mechanisms in the pathogenesis of sarcopenia [24,25]. Furthermore, the reduction of chronic, low-grade inflammation by use of nonsteroidal anti-inflammatory drugs has been demonstrated to have a protective effect against the loss of muscle mass and function in some animal and human studies [26,27]. In 2017, a meta-analysis reported that patients with sarcopenia had significantly higher levels of C-reactive protein (CRP) compared to controls [28]. Kwon et al. suggested that sarcopenia was significantly related to platelet and WBC counts, which are widely used as inflammatory parameters [29]. Our results showed that serum ALP levels were significantly associated with low muscle mass and were not inferior to WBC counts in predicting sarcopenia. However, one valid and unique biomarker for early detection and prediction of sarcopenia has not yet been identified due to its multifactorial pathogenesis [30]. Therefore, further studies on biomarkers with multifactorial approaches are needed.

The precise mechanism of how increased serum ALP levels are directly related to low skeletal muscle mass is unclear. A possible explanation may be the inflammatory regulation of ALP activity. ALP is known to catalyze the conversion of pro-inflammatory adenosine triphosphate to anti-inflammatory adenosine [31] and to reduce the level of bacterial endotoxin lipopolysaccharide through dephosphorylation [32,33]. Thus, increased ALP expression could be a cellular response to inflammatory or infectious stimuli. Previous experimental studies have demonstrated a link between ALP activity and various inflammatory cytokines, which are known to prompt muscle wasting and suppress muscle synthesis [28]. CRP and tumor necrosis factor-alpha (TNF-α) were associated with the induction of ALP activity in human osteoblasts and vascular smooth muscle cells (VSMCs) [34,35]. For example, TNF-α stimulated ALP expression in osteoblasts and calcium deposition in VSMCs [36]. In invasively ventilated critically ill patients, pulmonary ALP activity was positively correlated with levels of pro-inflammatory cytokines, such as interleukin (IL)-6 and IL-8 [37]. Further studies are needed to clarify the link between elevated serum ALP and sarcopenia through the inflammatory process.

This study has several limitations. First, we could not evaluate muscle strength and physical performance due to lack of data. In order to diagnose sarcopenia, it is essential to evaluate not only muscle mass but also muscle strength and physical performance. Further studies are needed to confirm the relationship between serum ALP levels and sarcopenia. Second, diseases that could elevate serum ALP levels, such as biliary obstruction, non-alcoholic fatty liver disease, hyperthyroidism, hyperparathyroidism, or sarcoidosis, were not fully excluded due to the nature of the secondary KNHANES dataset. Thus, some potential residual confounding factors were not taken into account in the multiple logistic regression analysis. In addition, medical conditions that could reduce serum ALP levels, such as postmenopausal hormone therapy or hypothyroidism, were not evaluated. Third, we could not determine which isoform of ALP, for example liver ALP or bone ALP, was associated with sarcopenia, because we did not measure ALP isoenzymes. Finally, the causal relationship between serum ALP levels and LSMI could not be identified due to the cross-sectional study design. Despite these weaknesses, to the best of our knowledge, this is the first study to examine the association between serum ALP levels and skeletal muscle mass, using a nationwide epidemiological data. Additional studies are necessary to determine the longitudinal effects of serum ALP levels on skeletal muscle mass, as well as muscle strength and physical performance.

## 5. Conclusions

Serum ALP levels, which may be a novel marker for inflammation, are found to be independently and positively related to low muscle mass in both men and women. Moreover, the predictive power of serum ALP levels for LMSI is not inferior to that of WBC counts. Our findings suggest the potential of serum ALP as an inflammatory marker, and motivate further prospective studies to demonstrate the validity of serum ALP as a predictive biomarker of sarcopenia.

## Figures and Tables

**Table 1:** Clinical characteristics of the study population.

	Men				Women			
Serum ALP level	T1 (≤200)	T2 (201–248)	T3 (≥249)	*p*	T1 (≤170)	T2 (171–224)	T3 (≥225)	*p*
Unweighted number, n	2409	2408	2354		2804	2859	2745	
Age, years	42.7 ± 0.4	42.2 ± 0.4	44.2 ± 0.5	0.001	37.9 ± 0.3	41.6 ± 0.4	48.5 ± 0.4	<0.001
Waist circumference, cm	83.7 ± 0.3	83.9 ± 0.3	84.6 ± 0.3	0.034	77.0 ± 0.2	80.8 ± 0.2	83.1 ± 0.2	<0.001
BMI, kg/cm^2^	24.0 ± 0.1	24.1 ± 0.1	24.2 ± 0.1	0.354	22.3 ± 0.1	23.1 ± 0.1	24.1 ± 0.1	<0.001
Mean blood pressure, mmHg	92.3 ± 0.4	93.2 ± 0.3	94.3 ± 0.3	<0.001	83.5 ± 0.3	86.5 ± 0.3	90.7 ± 0.3	<0.001
Fasting glucose, mg/dL	96.2 ± 0.5	97.0 ± 0.4	101.5 ± 0.7	<0.001	90.7 ± 0.3	93.1 ± 0.4	98.7 ± 0.6	<0.001
Total cholesterol, mg/dL	187.0 ± 1.0	187.0 ± 0.9	187.2 ± 1.1	0.980	179.4 ± 0.7	186.1 ± 0.6	189.6 ± 0.7	<0.001
Total calorie intake, kcal/day	2438.1 ± 26.8	2398.4 ± 27.0	2368.7 ± 27.0	0.170	1723.1 ± 17.7	1672.6 ± 15.9	1662.8 ± 16.7	0.030
Smoking status, % (SE)				<0.001				0.078
Current smoker	48.1 (1.5)	52.7 (1.5)	57.6 (1.5)		7.2 (0.7)	5.4 (0.6)	5.5 (0.6)	
Past smoker	23.9 (1.2)	20.4 (1.1)	20.7 (1.1)		2.8 (0.4)	2.8 (0.4)	2.1 (0.4)	
Never smoker	28.1 (1.4)	27.0 (1.3)	21.7 (1.2)		89.9 (0.8)	91.8 (0.7)	92.4 (0.7)	
Heavy alcohol drinker, % (SE)	62.4 (1.3)	62.5 (1.4)	57.8 (1.3)	0.014	45.3 (1.2)	37.0 (1.2)	26.0 (1.2)	<0.001
Regular exercise, % (SE)	27.1 (1.4)	26.5 (1.3)	28.0 (1.3)	0.692	23.0 (1.0)	21.9 (1.0)	23.5 (1.1)	0.506
Number of chronic diseases, % (SE)				<0.001				<0.001
0	87.2 (0.9)	85.9 (0.9)	80.0 (1.1)		93.3 (0.6)	89.8 (0.7)	81.2 (1.0)	
1	10.7 (0.9)	12.3 (0.8)	16.4 (1.0)		5.6 (0.6)	9.0 (0.7)	16.4 (0.9)	
≥2	2.2 (0.4)	1.8 (0.3)	3.6 (0.4)		1.1 (0.3)	1.2 (0.3)	2.4 (0.4)	
ASM, kg	23.0 ± 0.1	22.8 ± 0.1	22.5 ± 0.1	<0.001	14.6 ± 0.1	14.7 ± 0.1	14.7 ± 0.1	0.286
BMI-adjusted ASM	0.964 ± 0.004	0.952 ± 0.003	0.933 ± 0.004	<0.001	0.662 ± 0.002	0.643 ± 0.002	0.617 ± 0.003	<0.001

*p* values were derived from weighted generalized linear regression analysis for continuous variables and weighted chi-squared test for categorical variables. Abbreviations: ALP, alkaline phosphatase; BMI, body mass index; ASM, appendicular skeletal muscle mass SE, standard error.

**Table 2:** Weighted multiple logistic regression analysis between different tertiles of serum ALP level and LSMI in each sex.

Serum ALP Levels	T1	T2		T3		
		OR (95% CI)	*p*	OR (95% CI)	*p*	Overall *p*
Men						
Unadjusted	1 (reference)	1.05 (0.81–1.36)	0.736	1.72 (1.35–2.20)	<0.001	<0.001
Model 1	1 (reference)	1.06 (0.80–1.40)	0.684	1.60 (1.23–2.07)	0.001	<0.001
Model 2	1 (reference)	0.98 (0.70–1.37)	0.894	1.41 (1.04–1.91)	0.027	0.025
Women						
Unadjusted	1 (reference)	1.88 (1.35–2.60)	<0.001	3.84 (2.81–5.24)	<0.001	<0.001
Model 1	1 (reference)	1.18 (0.84–1.67)	0.341	1.42 (1.00–2.02)	0.049	0.123
Model 2	1 (reference)	1.16 (0.80–1.67)	0.441	1.45 (1.00–2.10)	0.049	0.107

Model 1: Adjusted for age and body mass index. Model 2: Adjusted for variables included in Model 1 plus smoking status, heavy alcohol drinker, physical activity, total calorie intake, and number of chronic diseases. Abbreviations: ALP, alkaline phosphatase; CI, confidence interval; LSMI, low skeletal muscle mass index; OR, odds ratio.

## Data Availability

The KNHANES data are publicly available through the KNHANES website (https://knhanes.kdca.go.kr/knhanes).
